# Fusarium Corneal Abscess: A Case Report

**DOI:** 10.7759/cureus.92523

**Published:** 2025-09-17

**Authors:** Najlae Ouamna, Yassine El Khalifa, Sarah Belghmaidi, Yasmine Rohi, Ibtissam Hajji, Abdeljalil Moutaouakil, Awatif El Hakkouni

**Affiliations:** 1 Department of Biology, Centre Hospitalo-Universitaire (CHU) Mohammed VI, Marrakech, MAR; 2 Parasitology-Mycology Laboratory, Mohammed VI University Hospital, Faculty of Medicine and Pharmacy-Cadi Ayyad University, Marrakech, MAR; 3 Department of Ophthalmology, Mohammed VI University Hospital, Faculty of Medicine and Pharmacy, Marrakech, MAR; 4 Parasitology-Mycology Laboratory, Mohammed VI University Hospital, Marrakech, MAR; 5 Department of Biology/Parasitology-Mycology Laboratory, Mohammed VI University Hospital, Faculty of Medicine and Pharmacy-Cadi Ayyad University, Marrakech, MAR

**Keywords:** case report, corneal abscess, fungal keratitis, fusarium, ophthalmic infection

## Abstract

*Fusarium* is a cosmopolitan fungus that can cause serious eye infections that threaten the functional prognosis of patients. Fusarium keratitis is the most serious, and its management remains laborious, requiring a multitude of therapeutic options ranging from medical treatment to surgical treatment. We report on the case of a 20-year-old patient who was diagnosed with a corneal mycotic *Fusarium* abscess grafted onto a healthy cornea. The diagnosis is made on a bundle of clinical and especially biological arguments by the demonstration of *Fusarium* on two occasions by direct examination and culture. The diagnosis must be considered in the presence of one or more risk factors; however, it must be considered even in their absence because, according to this case, *Fusarium* can graft onto a healthy cornea in immunocompetent individuals, based on clinical and biological data.

## Introduction

Fungal keratitis is a rare but often severe corneal infection. This is due to its invasive nature, which can lead to an ophthalmological emergency, namely corneal abscess, which threatens the functional prognosis of the eye but also the vital prognosis, particularly in immunocompromised patients susceptible to general dissemination of infections with a corneal starting point, generally caused by opportunistic agents on a damaged cornea [1]. Mycotic keratitis is mainly due to filamentous fungi or yeasts, with *Fusarium* being the most incriminated agent in the pathogenesis of keratomycosis [1].

*Fusarium* species are ubiquitous filamentous fungi found in soil and on plants and are a major cause of fungal keratitis, especially in tropical and subtropical regions, where they account for up to 47% of all fungal keratitis cases [2]. Infection usually follows trauma, particularly with plant material or contact lens use. However, cases in immunocompetent patients without clear risk factors are rare and underreported. Diagnostic challenges, intrinsic antifungal resistance, and frequent treatment failure make early detection and management essential. We report the case of a *Fusarium* corneal abscess in a healthy young adult with no known risk factors to highlight the clinical suspicion required and therapeutic considerations in atypical presentations.

## Case presentation

We report the case of a 20-year-old male with no particular pathological history, hospitalized for a corneal abscess. The patient presented with a painful, red right eye with decreased visual acuity that had been evolving for 15 days. The patient had no particular ophthalmological history; he did not wear contact lenses and did not describe any notion of trauma. The patient had no history of general pathology requiring the use of systemic immunosuppressive treatment, and during this episode, he resorted to self-medication with corticosteroids for eight days, then consulted a private doctor and was treated with oral acyclovir and local antibiotics for three days without any improvement before he was hospitalized in the ophthalmology department.

On admission, right eye visual acuity was "counting fingers" (CF); the left eye was 10/10. Pupillary reflexes were absent in the right eye and present in the left. Ocular tensions were normal bilaterally, assessed digitally due to the cornea being edematous. Examination of the ocular adnexa revealed blepharitis, conjunctival hyperemia (CHH), and mucopurulent discharge in the right eye, while no abnormalities were observed in the left eye (Table 1).

**Table 1:** Timeline of treatment and clinical progress

Day	Clinical Findings	Treatment	Outcome/Progression
Day 1	VA: MDM, IOP: Normal Axial corneal abscess 4 × 4.3 mm, Blepharitis, HHC, CPK, mucopurulent secretions Hypopyon present	Started hourly Voriconazole + Ceftazidime eye drops Abscess swab taken	Awaiting microbiology results
Days 2 t 3	Abscess enlarged to 5 × 4.5 mm, then 5.7 × 5.4 mm Same anterior segment findings as Day 1	Continued same treatment	Abscess progression
Day 5 to 6	VA: MDM, Axial abscess reduced to 3.6 × 2.7 mm Persistent HHC and hypopyon	Switched to Voriconazole intrastromal injections (3×, every 48h)	Clinical improvement noted
Day 10	VA: MDM, pre-perforative ulcer, abscess beginning to clean Corneal opacity forming	Continued Voriconazole fortified eye drops	Healing phase started
Day 11	VA: MDM, Corneal abscess with initial cleaning Persistent hypopyon IOP measured at 18 mmHg	—	AMG performed later that day
Day 12	VA: MDM, 360° chemosis, some secretions AMG in place HHC persists	Continued antifungals	Post-AMG observation
Follow-up	Epithelial healing, scar formation visible Candidate for corneal transplant	Started local corticosteroids + cyclosporine	Leucomatous axial scar, stable IOP

**Figure 1:**
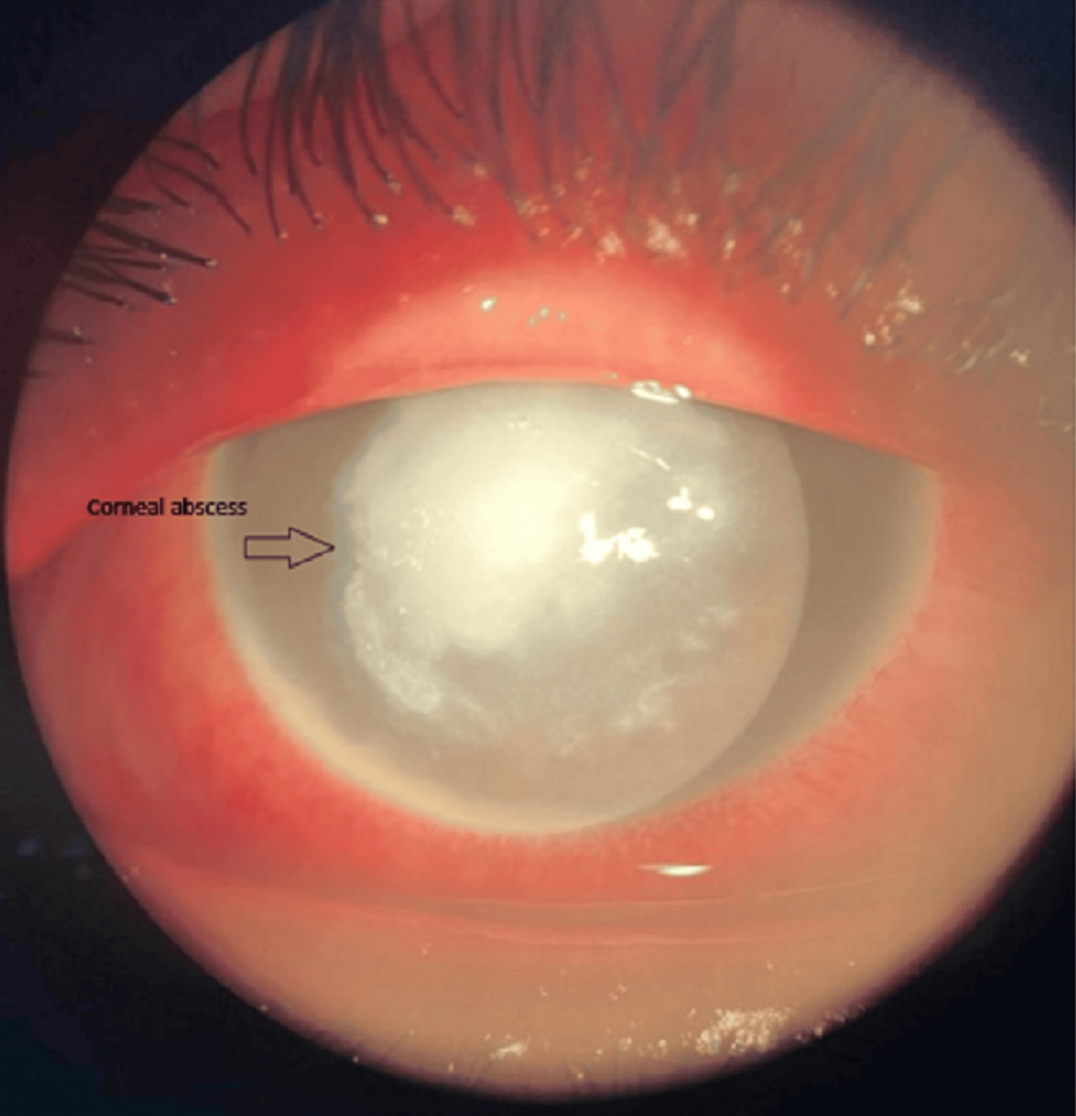
Anterior segment photograph showing a central corneal abscess with blurred and feathery margins.

**Figure 2:**
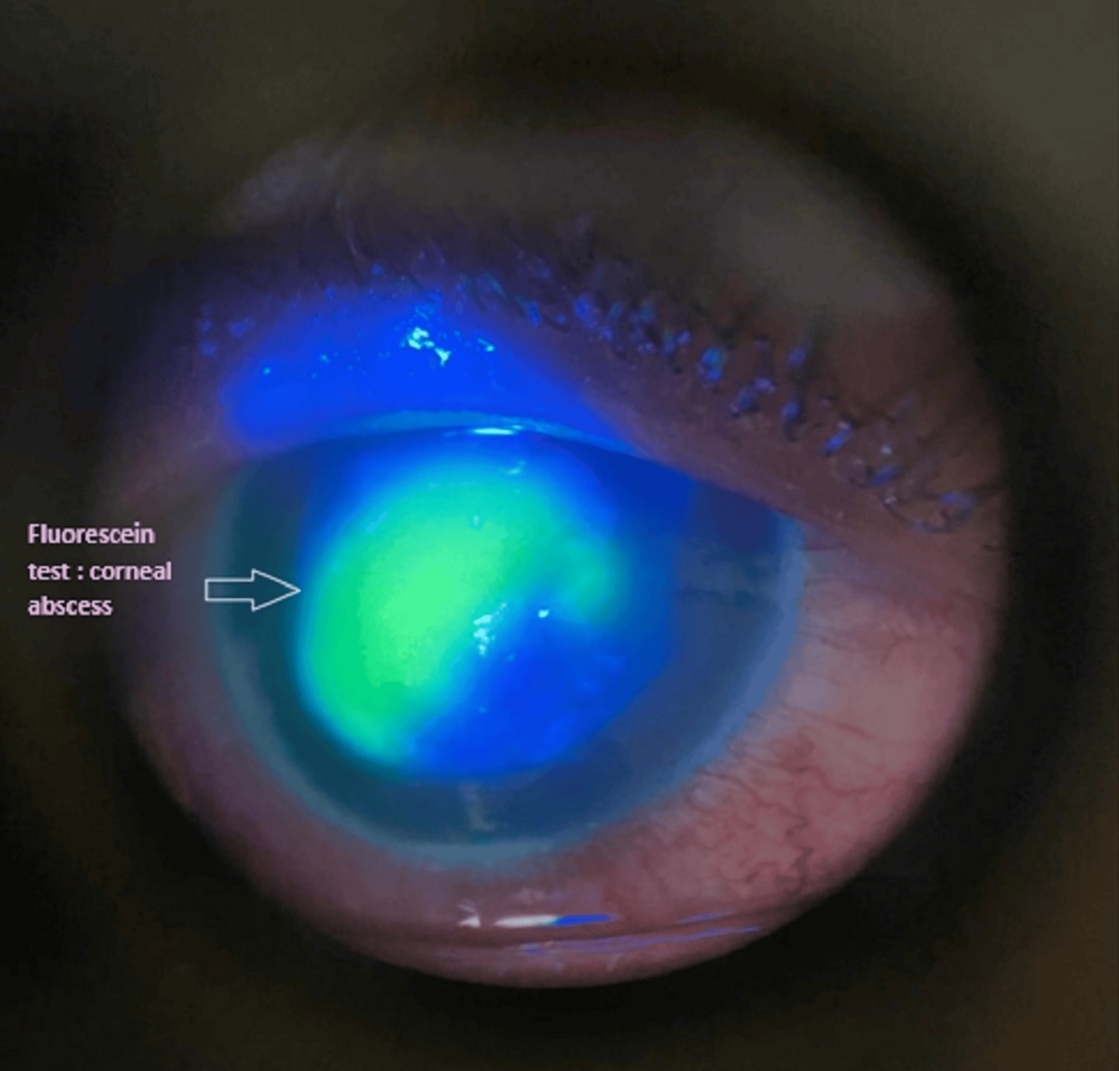
Anterior segment photograph of the right eye under cobalt blue illumination showing a central corneal abscess measuring approximately 4 × 4.3 mm, with fluorescein pooling over an epithelial defect. The lesion is dense, well-demarcated, and demonstrates a yellow-green fluorescence consistent with stromal infiltration and epithelial ulceration.

The corneal examination revealed an axial corneal abscess measuring 4*4.3 mm with a creamy appearance and corneal edema in the right eye (RE) (Figures 1, 2), in addition to a rough, dirty gray epithelial surface and corneal edema, while the cornea was clear and without abnormalities in the left (LE). A hypopyon was visible in the anterior chamber of the right eye with an invisible lens and an invisible fundus. B-mode ultrasound of the RE showed an anechoic vitreous and a flat retina.

**Figure 3:**
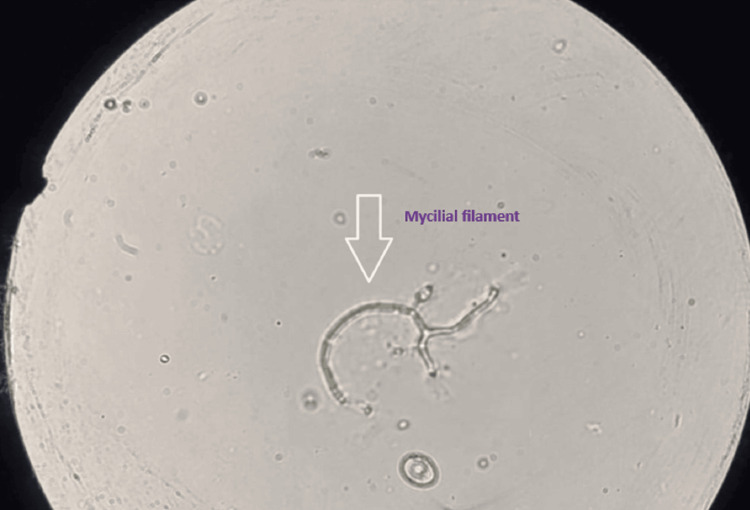
Direct microscopic examination of corneal scraping in fresh state showing hyaline, septate filament. Morphology is suggestive of Fusarium spp.

**Figure 4:**
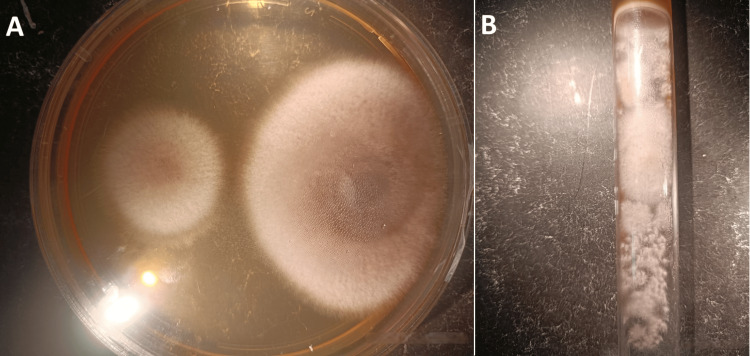
(A) Whitish downy colonies in the Petri dish on Sabouraud-chloramphenicol agar after 72 hours. (B) Whitish downy colonies in the tube on Sabouraud-chloramphenicol agar after 72 hours.

**Figure 5:**
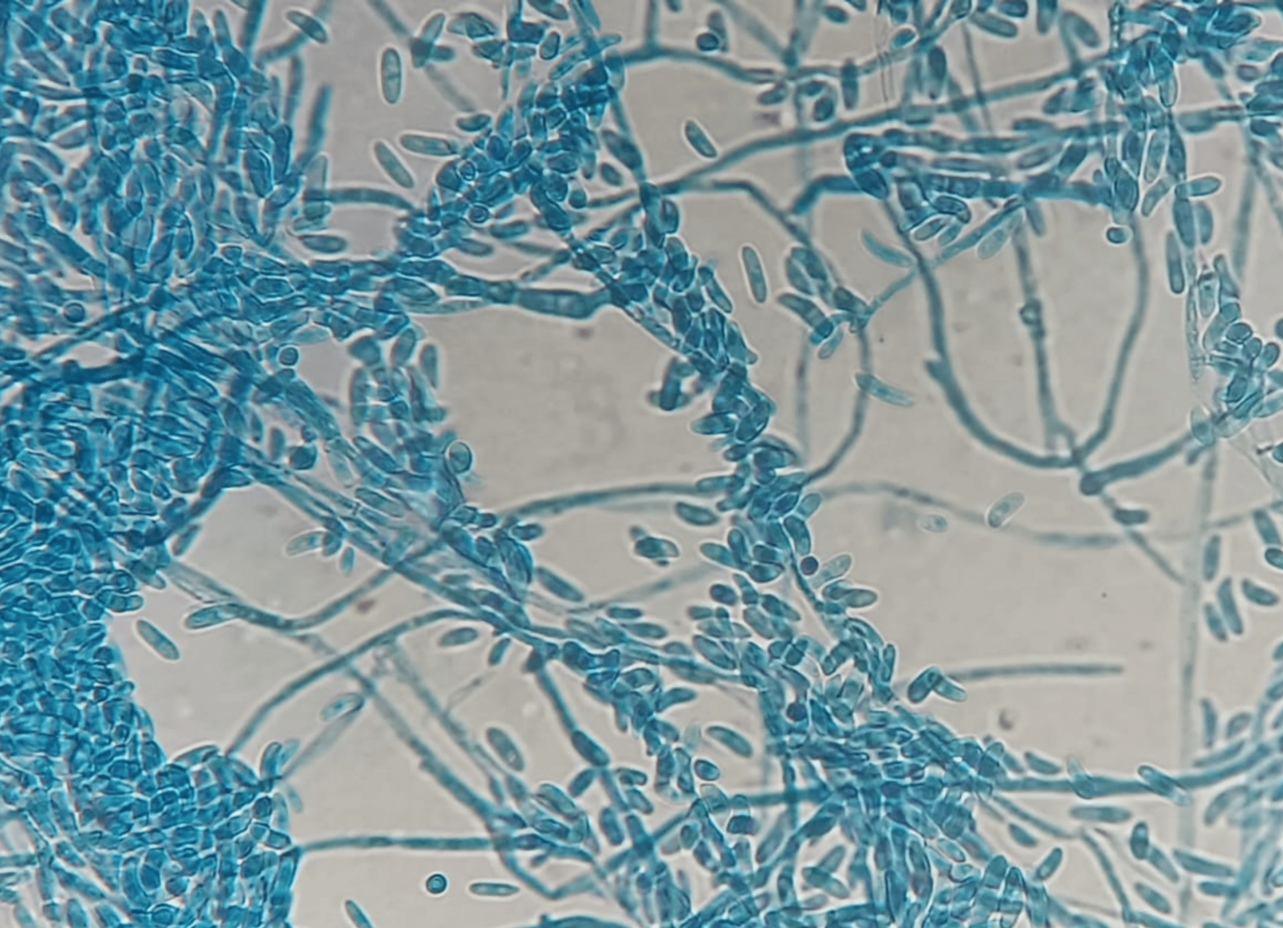
Microscopic appearance of Fusarium sp. under 400× magnification. Characteristic canoe-shaped macroconidia with multiple septa are visible.

Empirical anti-infective treatment was initiated urgently. It included the instillation of voriconazole and ceftazidime eye drops hourly for two days, then eight times a day. A swab of the abscess was taken before anti-infective treatment. Direct microscopy revealed mycelial filaments (Figure 3). Cultures on Sabouraud-chloramphenicol agar were positive with a growth time of 72 hours, showing colonies with a whitish downy appearance (Figures 4A, 4B). Identification of *Fusarium moniliforme* was possible using the flag technique (Figure 5). This result was checked and confirmed on a second sample (Table 1).

**Figure 6:**
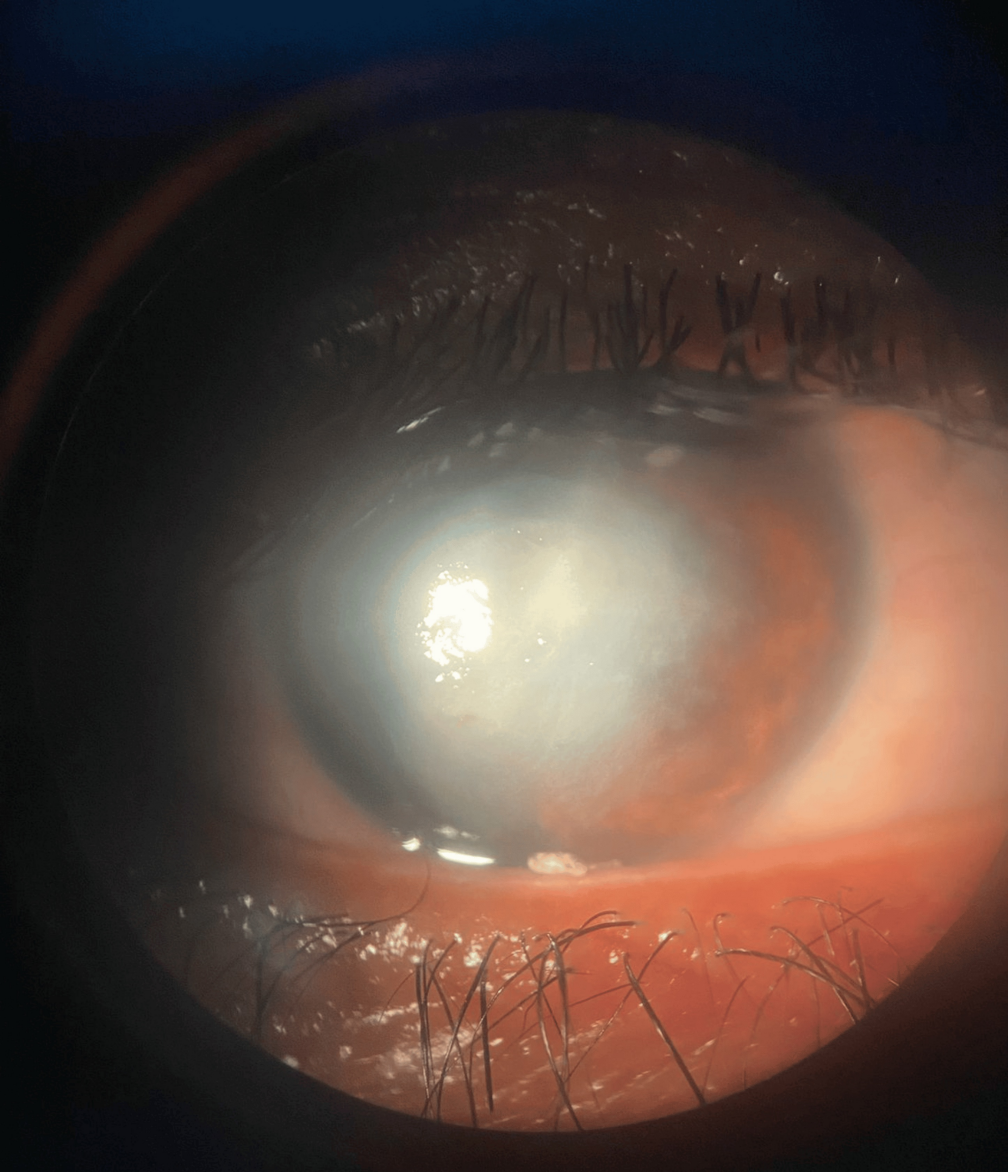
B-mode ultrasound of the RE after intrastromal instillation of voriconazole + corticosteroid therapy locally and cyclosporine RE: Right eye

In the absence of clinical improvement under voriconazole eye drops, three intrastromal injections of voriconazole were performed at 48-hour intervals. However, no clinical improvement was observed, and thinning of the cornea was noted, exposing the patient to the risk of perforation. Therefore, an amniotic membrane graft (AMG) was proposed to the patient and performed (Table 1). Voriconazole-fortified drops were continued for eight weeks. Local corticosteroids and cyclosporine were initiated once healing began to reduce inflammation and manage scarring (Figure 6). During the follow-up, intraocular pressure was measured at 18 mmHg using air-puff tonometry, and anterior chamber depth remained preserved despite the prior presence of a hypopyon.

## Discussion

Fusarium keratitis is a severe eye infection. It is the most common cause of blindness in tropical and subtropical areas [1]. Its prevalence in the world is difficult to specify; it is estimated at 1,051,787 cases per year [3].

*Fusarium* is a cosmopolitan soil-borne phytopathogen found in soil and plants (e.g., tomatoes, wheat, soybeans, corn) [1,2,4]. The genus *Fusarium* includes 200 species, divided into 10 species complexes, which are highly pathogenic to humans: *F. solani*, *F. oxysporum*, *F. fujikuroi*, *F. incarnatum-equiseti*, *F. clamydosporum*, *F. dimerum*, *F. sambucinum*, *F. concolor,* and *F. lateritium*. *F. solani* complex is the most incriminated and the most virulent in human pathogenesis [2].

The affinity of *Fusarium* for humid environments has been reported in several studies, and the hypothesis of water contamination must be taken into account, particularly in hospital environments housing immunocompromised patients [2-5].

There appears to be a strong geographical influence on the occurrence of different forms of mycotic keratitis. The proportion of corneal ulcers caused by filamentous fungi increases towards tropical and subtropical areas [1].

*Fusarium* has long been known for its pathogenic role in superficial infections (cutaneous, keratitis, and onyxis). However, it is now also one of the emerging fungal pathogens responsible for invasive opportunistic infections that are life-threatening in immunocompromised patients [2].

The pathogenesis of Fusarium keratitis depends on factors related to the host immune response and the characteristics of *Fusarium*. Its capacity to produce mycotoxins such as fumonisin B1, as well as its angioinvasive power and its ability to produce biofilm in the presence of foreign materials such as contact lenses or catheters, was elucidated in recent studies [3]. For the host, once *Fusarium* invades the corneal stroma, innate immune cells such as macrophages, neutrophils, and dendritic cells provide host defense [1]. A cascade of inflammatory events will occur, and there is a release of pro-inflammatory factors such as interleukins 1,6,17, and 8 [1-4]. The inflammatory reaction caused by these fungi depends on their replication, the secretion of mycotoxins and proteolytic enzymes, and fungal antigens [1].

Fusarium keratitis typically occurs on a damaged cornea with compromised ocular surface defenses and is frequently observed following corneal trauma from plant material, exposure to dust, or in individuals wearing hydrophilic contact lenses [1]. According to a 10-year review at a tertiary eye care center in South India, *Fusarium* accounted for up to 43% of fungal keratitis cases, the vast majority of which were associated with trauma or predisposing conditions [5]. However, our patient had no identifiable corneal lesions or systemic risk factors. A *Fusarium* infection developed on an otherwise healthy cornea in an immunocompetent individual, an atypical and rarely reported presentation. To our knowledge, only a few such cases have been described in the literature, highlighting a significant gap in our understanding of Fusarium keratitis in patients without known risk factors [2].

In Fusarium keratitis, clinical signs are not very suggestive: the eye is red, painful, and watery, with the presence of photophobia, blepharospasm, and reduced visual acuity, reflecting the clinical presentation of our patient.

The diagnosis should be considered in the presence of any slowly progressive corneal ulceration because progression occurs towards endophthalmitis, which may require enucleation of the eyeball [6].

However, the diagnosis of Fusarium keratitis cannot be made only on clinical presentation. It is therefore necessary to highlight *Fusarium* by sampling the site of infection, and in this case, it is the cornea. A direct examination is thus conducted using May Grunwald Giemsa MGG stain, but the latter does not allow differentiation between the filaments of *Aspergillus* and those of *Fusarium*, which are hyaline, septate, and fine but irregular (Figure 3). This requires the isolation and identification of the fungus by culture [3-6]. In culture, *Fusarium* is easily isolated 48 to 72 hours on Sabouraud agar without cycloheximide at 30-35°. Isolatedenus can be identified by the presence of typical multicellular macroconidia. Species identification is difficult and may require molecular methods or even MALDI-TOF [6].

There is no serodiagnosis; the search for the galactomannan antigen on the serum is often positive (cross-reaction with aspergillosis), as well as the search for the BD-glucan antigen [1-3,6].

The genus *Fusarium* is resistant to natamycin, amphotericin B, and voriconazole. There is an intrinsic resistance that already exists without exposure to antifungals, in addition to the possibility of having acquired resistance, which is common in medical practice [2]. Hence, the difficulty of treatment and the frequency of therapeutic failures.

Natamycin has demonstrated antifungal activity against *Fusarium* species, particularly *F. solani*, and remains the only topical antifungal approved for ocular use. Several studies, including minimum inhibitory concentration (MIC) evaluations, have shown that some *Fusarium* isolates are susceptible to natamycin, with MIC values ranging between 4 and 8 µg/mL, although higher values have also been reported depending on the strain [2-7]. Despite this, natamycin has limited stromal penetration, which may restrict its effectiveness in deeper keratitis. For this reason, and especially in refractory cases, natamycin is often combined with voriconazole, which offers better intraocular tissue penetration. This combination has shown enhanced efficacy, broader antifungal coverage, and reduced inflammatory response through suppression of fungal biofilm formation [2-7].

On the other hand, the combination of natamycin and amphotericin B has not demonstrated a consistent synergistic effect, possibly due to overlapping mechanisms of action, as both target ergosterol in the fungal cell membrane [3]. Therefore, while natamycin may retain antifungal activity against certain Fusarium species, particularly in superficial infections, combination therapy remains the preferred approach in cases of extensive or resistant keratitis.

In refractory cases of *Fusarium* corneal ulcer, the indication of intrastromal injection of antifungals takes its place, with the aim of increasing their concentrations in the affected tissue; however, the results on the clinical and experimental level remain non-exclusive [2].

When other therapeutic options do not give good results, amniotic membrane transplantation (AMT) is an alternative to preserve the functional prognosis of the patient's eye [2]. AMT provides an avascular and acellular structure, playing the role of a basement membrane limiting inflammatory phenomena and corneal vascularization and facilitating healing. After the transplantation, the patient is monitored closely for signs of healing and any potential complications. Corticosteroids and cyclosporine may be prescribed to support the healing process and reduce inflammation [8].

## Conclusions

Fusarium keratitis is a severe fungal infection that compromises the functional prognosis of the eye and can lead to blindness or other ophthalmologic sequelae. It most commonly occurs in patients with well-documented risk factors, such as ocular trauma, contact lens wear, or preexisting corneal disease. However, as demonstrated in this case, *Fusarium* can occasionally infect a structurally healthy cornea in immunocompetent individuals, which remains a rare and underreported presentation in the literature. Early diagnostic vigilance is therefore essential, even in atypical cases lacking conventional risk factors. Therapeutic management remains challenging due to the intrinsic resistance of *Fusarium spp.* to multiple antifungal agents, including natamycin, amphotericin B, and voriconazole. This case highlights the potential benefit of combining natamycin and voriconazole, which together may offer enhanced efficacy against this highly resistant organism, especially when initiated promptly and monitored closely for clinical response.
